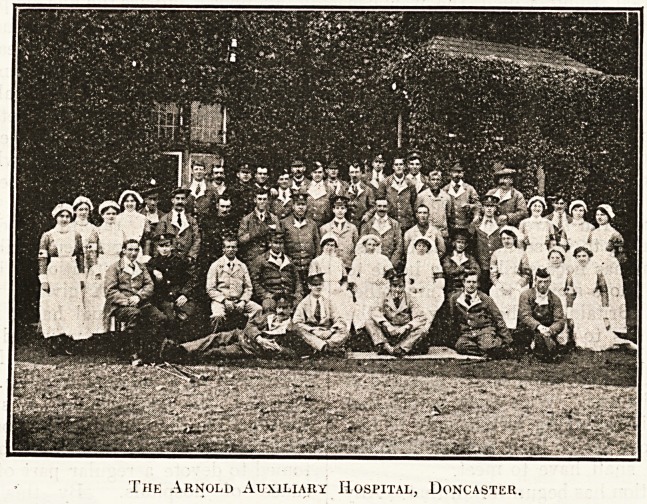# The Record of the Arnold Hospital, Doncaster

**Published:** 1915-12-18

**Authors:** 


### A YEAR'S WORK AT AN AUXILIARY HOSPITAL.

### The Record of the Arnold Hospital, Doncaster.

We publish the following account of the work of the Arnold Auxiliary Hospital, Doncaster, because its work, as celebrated in its first anniversary, on Thursday, December 9, is typical of the usefulness of these institutions, which have not generally received the publicity which the best of them deserve. We hope the publication of this record may encourage other institutions, whose workers are alive to the interesting material provided by these new institutions, to set forth what they have accomplished, and send us the account, thus throwing side-lights on this new department of hospital work, and thereby awakening wider general interest in it.

Originally the Arnold Hospital consisted simply of a spacious suburban house, standing in grounds charmingly laid out, and for several years the residence of Mr. W. Sayles Arnold, a well-known public works contractor, who placed it at the disposal of the V.A.D. for an indefinite period, free of rent. For some months the Detachment worked under crowded conditions, the house proving much less than adequate to accommodate the convalescents whom the authorities at the Sheffield Base Hospital wished to send along. From the first, however, public support in money and kind was generously forthcoming, and when it was decided to build an annexe in the grounds, and so more than double the cot accommodation, there was no anxiety on the score of finances. Nearly £350 was raised by a local Flag Day alone, and this was more than sufficient to cover the entire cost of the extension. The annexe was opened in July by Viscountess Halifax, vice-president of the 14th District V.A.D.

The Arnold Auxiliary Hospital, Doncaster.

"Arnold's" now accommodates fifty soldiers, and is generally full. Since it was opened no fewer than 519 convalescents have passed through the hands of the staff. An interesting event in the year was the reception by a Canadian soldier, Sapper Duncan, while recuperating at "Arnold's," of the Distinguished Conduct Medal gained by him in France. Surgeon-Colonel Connell, of the Sheffield Base Hospital, has praised the condition of body and spirits in which men report themselves after their course of convalescence at Doncaster.

The Arnold Hospital is happy in the possession of an excellent staff, from the Commandant, Mrs. W. H. Pickering—widow of the late Mr. W. H. Pickering, H.M. Divisional Inspector of Mines, who died a hero's death in the Cadeby Colliery disaster three years ago—downward; Miss Smith as quartermaster. Mrs. Rogers in charge of the catering department, and Sister Annie, of the Victoria Nursing Home, as superintendent of the nursing staff, are excellent V.A.D. workers; and in Lieut.-Colonel Stevenson the hospital possesses a medical officer of long service in the Regular Army. Starting with a membership of less than a score, the Detachment has now a regular hospital staff of nearly forty, and by means of emergency classes organised last winter some 130 young Doncaster ladies were able to pass the St. John Ambulance Association tests. Several nurses who received their first practical training at "Arnold's" are now working in the regular military hospitals at home and abroad.

## Figures and Tables

**Figure f1:**